# Cortical origin of Up state onsets and offsets in anesthetized rats

**DOI:** 10.1186/1471-2202-14-S1-P341

**Published:** 2013-07-08

**Authors:** Maria Perez-Zabalza, Maurizio Mattia, Nuria Tort, Maria V Sanchez-Vives

**Affiliations:** 1 IDIBAPS, c/. Rosselló, 149 – 153, 08036 – Barcelona, Spain; 2 Istituto Superiore di Sanità, Rome, Italy; 3 ICREA, Passeig Lluís Companys, 23, 08010 Barcelona - Spain

## 

Slow rhythms (< 1 Hz) where Up states of sustained neural activity alternate with almost quiescent Down states, are similarly expressed by mammal brains during slow-wave-sleep (SWS) and under anesthesia [1]. Although much effort has been devoted to uncover the physiological origin of such slow oscillations (SO), it is still debated whether Up state onsets in this quasi-periodic bouncing of neural activity are driven by cortico-cortical synaptic interactions [1-3], or if they are due to the interplay between thalamic nuclei and cerebral cortex [4,5]. We addressed this issue probing neuronal and synaptic activity across all layers of primary visual cortex (V1) of rats, which showed stable Up/Down SO when anesthetized with ketamine and medetomidine.

During Up states, maximum multi-unit activity (MUA) was found at a depth of 880 ± 80 μm (mean ± SD, n = 14) from cortical surface, corresponding to upper layer 5. To be able to classify events as supra/infragranular and thus to accurately identify layer 4 position, we resorted to a classical current source density (CSD) analysis looking at the earliest synaptic activation following visual stimulation [6]. This, together with a principal component analysis (PCA), allowed us to characterize Up state onsets at high spatiotemporal resolution as a stereotyped sequence of events. Firstly, in deep layers (1260 ± 80 μm, mean ± SD, n = 14) a CSD sink appeared highlighting the arrival of synaptic input, always followed after 10 ms by a MUA onset at same depths. After a variable delay between 0 and 40 ms, MUA increased in upper layer 5 and propagated to supragranular layers. Interestingly, such upward spreading of activity from deep to more superficial layers was not altered by the inactivation of visual thalamus obtained injecting TTX in LGN (lateral geniculate nucleus). Downward transitions from Up to Down states, showed a different timing of MUA offsets across layers. We found both positive and negative time lags between MUA inactivation of upper layer 5 and deeper layers, highlighting only a weak correlation between these two events. In layer 5-6, CSD sinks followed with a rather precise delay of 50 ms MUA offsets at the same depth, supporting the hypothesis of a network whose preferred state is the quiescent one, despite a steady synaptic input continue to be received. Even for downward transitions the chain of events was unchanged injecting TTX in the thalamus.

The collected evidence for both Up state initiation and termination unambiguously reserves for the thalamus a marginal role, pointing out cortico-cortical connectivity as the main drive for Up/Down SO. Such cortico-cortical chat takes place in a state-dependent manner.
